# A protocol integrating remote patient monitoring patient reported outcomes and cardiovascular biomarkers

**DOI:** 10.1038/s41746-019-0145-6

**Published:** 2019-09-03

**Authors:** Chrisandra Shufelt, Eldin Dzubur, Sandy Joung, Garth Fuller, Kelly N. Mouapi, Irene Van Den Broek, Mayra Lopez, Shivani Dhawan, Corey W. Arnold, William Speier, Mitra Mastali, Qin Fu, Jennifer E. Van Eyk, Brennan Spiegel, C. Noel Bairey Merz

**Affiliations:** 1 Barbra Streisand Women’s Heart Center, Cedars-Sinai Smidt Heart Institute, Cedars-Sinai Medical Center, Los Angeles, CA USA; 2 Cedars-Sinai Center for Outcomes Research and Education (CS-CORE), Los Angeles, CA USA; 3 Advanced Clinical Biosystems Research Institute, Cedars-Sinai Smidt Heart Institute, Cedars-Sinai Medical Center, Los Angeles, CA USA; 4 Medical Imaging and Informatics Group, University of California, Los Angeles, CA USA

**Keywords:** Predictive markers, Cardiovascular diseases

## Abstract

We describe the protocol, design, and methodology of the Prediction, Risk, and Evaluation of Major Adverse Cardiac Events (PRE-MACE) study as a multicomponent remote patient monitoring in cardiology. Using biosensor, biomarkers, and patient-reported outcomes in participants with stable ischemic heart disease, the PRE-MACE study is designed to measure cross-sectional correlations and establish the ability of remote monitoring to predict major adverse cardiovascular event (MACE) biomarkers and incident MACE at baseline and 12-month follow-up. It will further assess the adherence and cost-effectiveness of remote monitoring and blood sampling over the initial months. Despite medication and lifestyle changes, patients with cardiovascular disease can experience MACE due to undertreatment, poor adherence, or failure to recognize clinical or biochemical changes that presage MACE. Identifying patients using remote monitoring to detect MACE forerunners has potential to improve outcomes, avoid MACE, and reduce resource utilization. Data collection will include: (1) continuous remote monitoring using wearable biosensors; (2) biomarker measurements using plasma and at-home micro-sampling blood collection; and (3) patient-reported outcomes to monitor perceived stress, anxiety, depression, and health-related quality of life. Two hundred participants will be followed for 90 days with a subset (*n* = 80) monitored for 180 days. All participants will be followed up for MACE at 12 months.The PRE-MACE study will utilize remote monitoring with biosensors, biomarkers, and patient-reported outcomes to identify intermediate biomarkers of MACE in patients with stable ischemic heart disease. If shown to be effective, this intervention can be utilized between health visits to predict MACE and reduce financial impact of MACE.

## Introduction

Ischemic heart disease (IHD) is the leading cause of death in the United States in men and women across all ethnicities.^1^ Although lifestyle risk factors associated with IHD such as smoking, physical inactivity, poor nutrition, and obesity are well understood, interventions designed to induce behavior changes suffer from poor adherence and behavior substitutions. For individuals already diagnosed with IHD, secondary prevention focuses on controlling blood pressure, cholesterol, and often diabetes, which is managed within the confines of a health care provider’s clinical setting. Furthermore, algorithmic tools to predict cardiovascular events, such as the atherosclerosis cardiovascular disease risk calculator that considers age, sex, race, blood cholesterol, blood pressure, diabetes, and smoking status, are often only performed in hospital or clinic visits. Identifying early markers predictive for those individuals that are at an increased risk for developing cardiovascular events between visits to capture day-to-day or month-to-month changes in symptomology, biochemical biomarkers, or psychosocial behavior may potentially prevent catastrophic situations, improve access to care, and reduce resource utilization.

With advances in digital medicine, there are now opportunities to perform continuous remote patient monitoring of health outcomes using experience sampling methodologies such as patient-reported outcomes (PROs) and ambulatory physiological measures such as heart-rate and activity, both to improve ecological validity and obtain continuous insight of participants in real-time situations.^2,3^ The acceptance and availability of consumer-grade biosensors with clinical capabilities continue to improve, driven by advancements in technology and reduction in cost to patients. However, despite a trend toward ubiquity of health-sensing devices among consumers, the voluntary contribution of collected information to medical records remains challenging, even among health systems with dedicated smartphone applications.^4^ In one large-scale initiative inviting patients to share personal fitness tracker data with their providers, only 0.8% of patients opted to connect their wearable data to the electronic health record.^4^ Self-reported outcomes and passive sensors may not provide a complete clinical profile of a patient as compared to biomarkers. Moreover, the predictive ability of passive data may be limited by small sample size at the participant level, as a result of relatively infrequent events.

Circulating biochemical biomarkers also play an important role in predicting risk of cardiovascular events and should be considered alongside remote monitoring of PROs and biometrics. Among the important biomarkers for potential MACE prediction are high sensitivity C-reactive protein (hsCRP), N-terminal pro-brain natriuretic peptide (NT-proBNP), and ultra-high sensitivity cardiac-specific Troponin I (u-hs-cTnI).^5^ Furthermore, the use of micro-sampling devices to collect small volumes of blood remotely away from health care facilities could provide additional circulating biomarker information in a semi-continuous manner, expanding the capacity of remote patient monitoring beyond biometrics and psychometrics to now include home-based biochemical sampling.^6^

Funded by the California Initiative for the Advancement of Precision Medicine (CIAPM), a State-wide public program designed to spur pragmatic precision medicine research for common diseases, the Prediction, Risk, and Evaluation of Major Adverse Cardiac Events (PRE-MACE) study is a multimethod, longitudinal, prospective cohort study design in 200 patients with stable IHD. This study is designed to evaluate feasibility regarding adherence and pilot with regard to value using remote monitoring with biosensors, biomarkers, and PROs. The overall goals of the study are: (1) measure cross-sectional correlations between established major adverse cardiovascular event (MACE) intermediate biomarkers; (2) explore the longitudinal ability of remote physiological and biochemical monitoring to predict changes in MACE intermediate biomarkers; (3) explore the ability of remote monitoring and biomarkers to predict incident MACE at baseline and over 12-month follow-up; (4) carry out discovery proteomics and lipidomics to expand potential circulating predictive MACE biomarkers, and (5) estimate the cost-effectiveness and budget impact of remote monitoring for potential MACE biomarkers and MACE itself. Here, we describe the protocol design and methodology of the PRE-MACE study as an exemplar of a multicomponent remote patient monitoring study in cardiology.

## Discussion

Despite lifesaving medications, such as cholesterol-lowering statins, blood pressure medications, intensive antiplatelet therapy, and revascularization strategies, many stable IHD patients progress to MACE due to undertreatment, poor adherence to treatment, or failure to recognize clinical or biochemical changes that occur prior to MACE in order to deploy intervention. Patients with stable IHD may also develop precursor myocardial injury that is not easily detected with standard monitoring yet may progress to MACE if not recognized, leading to potentially fatal outcomes. If remote monitoring and predictive markers are able to identify subtle signals that predict MACE, then more precise, timely, and cost-effective interventions can be deployed to improve outcomes, avoid catastrophic events, improve care access for large outpatient populations, and reduce resource utilization.

The PRE-MACE study is designed to evaluate feasibility regarding adherence and pilot remote monitoring with biosensors, biomarkers, and PROs. This study will collect foundational data to answer fundamental questions about the predictive validity of remote monitoring for MACE and provide preliminary data for future studies. It will be a next-generation digital health monitoring protocol that leverages advances in mobile health technologies, including remote monitoring of wearable biometrics, app-based psychometrics, and home-based biochemical biomarker collection. Using a combination of a wearable activity tracker, an FDA-cleared remote ECG monitor, a smartphone app for PRO monitoring, and the Mitra® home microsampling device coupled to apolipoprotein quantification, this protocol seeks to expand the capabilities of digital health in an effort to find “signals in the noise” that may presage MACE. Although the study is focused on stable IHD, we view it as a model for conducting population-based precision health remote monitoring research for any chronic disease.

In addition, the success of digital health will depend not only on improving efficacy and effectiveness of care, but also ensuring cost-effective healthcare delivery. Modern digital health protocols should, where possible, consider health economic considerations early in the process of designing and testing new technologies and devices. Of note, the State of California requires health economic modeling among all grant recipients under its CIAPM program. For the current protocol, we will formally project the cost-effectiveness and budget impact of future remote patient monitoring programs that assess physiological and biochemical signals for stable IHD. These hypothesis-generating models will incorporate data from the study itself, including costs and consequences of the remote monitoring program, and couple the results with evidence from the literature to estimate the potential cost-effectiveness of the program under varying scenarios and budget constraints. This process will ultimately help health systems and policymakers to determine whether to support future programs and will assist researchers in identifying threshold for cost-effectiveness.

This State-sponsored CIAPM project will generate a wide range of data across wearable biometric, app-based psychometric, and home-based biochemical biomarker platforms. This protocol will serve as the foundation for future publications emanating from the study. It is also our hope that publishing this protocol as a standalone document offers a model for conducting multidisciplinary digital health research across diverse data collection platforms.

## Methods/design

### Recruitment and inclusion/exclusion criteria

Participants with stable IHD and intermediate risk for MACE, defined as an annualized MACE risk of 10–13%,^7^ will be recruited from a large, academic hospital-based cardiac rehabilitation center, a tertiary care women’s heart center, and physician referral. Additional recruitment strategies will include using pre-existing research registries of patients with stable IHD. Inclusion criteria include: 18 years of age or older, current diagnosis or history of ischemic heart disease, owning or having access to a smartphone or device, and willing to return for required follow-up visits. Exclusion criteria include: symptoms or signs of acute coronary syndrome and/or heart failure (Class III/Class IV), planned revascularization or valve surgery, comorbidity that precludes 12-month survival, or history of psychiatric disorders or substance abuse. An “intermediate” risk group is selected because they account for the majority of MACE, compared to the less frequent “high” risk participants who are typically monitored and treated by existing strategies, and the “low” risk participants who represent a lower population burden. All participants will provide signed informed consent for baseline evaluations and follow-up by using forms and procedures in accordance with institutional guidelines and approved by the institutional review board.

The study will recruit 200 participants based on sample size calculation for the aims 1 and 2 analyses featuring multivariable linear regression, treating the predictor variables as fixed. The Stata 13 program with the powerreg commend was used to power the study around *F*-tests of whether the β’s for PROs, remote patient monitoring data, and biomarkers are simultaneously zero while controlling for confounding variables. Setting alpha to 0.0001 and seeking 80% power to detect an improvement in *R*-squared of 0.025 when comparing existing models to models augmented by study data.

### Remote patient activity monitoring

During a baseline visit, participants will be provided a Fitbit Charge 2 device (Fitbit Inc., San Francisco, CA, USA), a wrist-worn device that tracks heart rate and activity along with in-person training. Monitoring with the Fitbit will be continuous, only interrupted by bathing, swimming, other activities involving water, or charging the device. The Fitbit Charge 2 is a consumer device with an embedded tri-axial accelerometer to track activity and a photoplethysmography (PPG) sensor to detect active heart rate and resting heart rate.^8^ PPG and accelerometry are also used to determine, whether an individual is asleep and whether or not they are actively wearing the device. Fitbit data will be continuously synced and uploaded into the Fitabase system which staff will track for return of results and compliance. When noted, study staff will contact the participant to query compliance. Given the motivated population, we anticipate a target compliance of over 80%. Previously published data has revelated over 90% compliance with the Fitbit device using this population.^9^ A subset of *n* = 80 (40%) participants will be enrolled in an extension cohort, which will include an additional 90-day monitoring period using biosensors, biomarkers, and patient-reported outcomes for a total of 6 months.

A subset (153, 77%) of participants will also receive the AliveCor KardiaMobile device (AliveCor, Mountain View, CA) and will be asked to monitor a rhythm electrocardiogram stripe weekly or if they experience any cardiac symptoms. AliveCor KardiaMobile is an FDA-cleared, single-channel electrocardiogram rhythm strip that detects the presence of atrial fibrillation and normal sinus rhythm.^10^ Participants may record symptoms experienced during the session through a smartphone application, while simultaneously placing index fingers on the electrodes of the device for 30 seconds. The AliveCor KardiaMobile emits a high-frequency signal containing the heart rhythm data that is detected by the AliveCor smartphone application. This information is then transmitted to AliveCor and interpreted using machine learning algorithms and communicated back to the participant as either normal or potentially abnormal heart rhythm. The AliveCor KardiaMobile device has been validated in clinical populations diagnosed with arrhythmias.^11^ Table 1 provides an overview of testing, data collection, and frequency of the PRE-MACE protocol.

**Table 1:** PRE-MACE protocol data collection

Testing and data collection	Frequency over 90-day period
Remote monitoring	
Fitbit Charge 2	Continuous
KardiaMobile AliveCor	Weekly
MACE intermediate biomarkers	
u-hs-cTnI	Day 0 (Baseline), Day 90 (Exit)
NT-proBNP	Baseline, Exit
hsCRP	Baseline, Exit
Mitra microsampling device (Apolipoprotein panel: A-I, A-II, A-IV, B, C-I, C-II, C-III, E, J)	Baseline, Day 30, Day 60, Exit with subset Day 90 and 120
Proteomics discovery	Baseline
Patient reported outcomes	
PROMIS questionnaire	Baseline, Weekly
Depression	Baseline, Weekly
Emotional distress/anxiety	Baseline, Weekly
Fatigue	Baseline, Weekly
Physical function	Baseline, Weekly
Sleep disturbance	Baseline, Weekly
Social isolation	Baseline, Weekly
Global mental health	Baseline, Weekly
Global physical health	Baseline, Weekly
Kansas City Cardiomyopathy Questionnaire (KCCQ)	Baseline, Bimonthly
Seattle Angina Questionnaire (SAQ)	Baseline, Monthly

### Biochemical biomarker monitoring

At baseline (day 0) and exit (day 90) enrolled participants will complete a venous plasma draw for the MACE intermediate biomarkers, hsCRP, NT-proBNP, u-hs-cTnI. Both hsCRP and NT-proBNP will be measured by the Immunochemical Core at Mayo Clinic on a Cobas c311 (hsCRP) and a Cobas e411 (NT-proBNP) chemical analyzer (both Roche Diagnostics, Indianapolis. IN). u-hs-cTnI will be measured using the Quanterix assay (Quanterix Corporation, Lexington, MA) on the Simoa HD-1 Analyzer, a highly sensitive and fully automated ELISA platform, in duplicate at the Cedars-Sinai Protein Quantitation Core.^12,13^ Any sample with the percent coefficient of variation (%CV) higher than 20% for u-hs-cTnI will be repeated with appropriate calibrators and controls.

Remote biochemical biomarker monitoring will occur using the Mitra® micro-sampling device (Neoteryx, Torrance, CA), an FDA-listed class 1 device (D254956) that allows remote, longitudinal, and volumetric blood sample collection using a simple finger prick.^14^ The Mitra device has an absorbent polymeric tip that wicks up a fixed volume (10 µL) of blood and allows sample heterogeneity and overcomes hematocrit bias issues associated with dried blood spots.^19^ At baseline (day 0) and time of exit (day 90), four Mitra tips will be filled onsite using the finger prick blood collection. Participants will be provided two additional kits for remote blood collection after one month (day 30) and two months (day 60). At the second onsite visit (day 90) a subset of up to 40 individuals will be provided three additional kits to be returned at day 120, 150, and 180, while another subset of up to 40 will be provided one additional day 180 kit. Automated email reminders or push notifications for home Mitra blood collection (day 30 and day 60 and for the extended subset, day 120, 150 and 180) will be sent to participants one day prior to scheduled draw. The option to obtain blood collection onsite at the hospital with help from the study staff will be made available to each participant.

Apolipoproteins (A-I, A-II, A-IV, B, C-I, C-II, C-III, E, J), human serum albumin, and hemoglobin will be extracted, denatured, reduced, alkylated, and digested from the Mitra tips using a previously optimized and automated workflow on the Biomek NXP Workstation (Beckman Coulter).^6,15,16^ A targeted multiple reaction monitoring mass spectrometry approach will be used for protein quantification on the 6500 Triple Quadrupole mass spectrometer (SCIEX) with stable isotope labeled peptides spiked into each sample similar to our previous protocols.^6,16^ (see Table 2 for the qualifier and quantifier peptides).

**Table 2:** Protein composition measured from the Mitra microsampling devices

Protein name	Abbreviation	Peptide sequence used in assay
Apolipoprotein A-I	apoA1	AKPALEDLR
		DLATVYVDVLK
Apolipoprotein A-II	apoA2	SPELQAEAK?
		EQLTPLIK
Apolipoprotein A-IV	apoA4	ISASAEELR
		LLPHANEVSQK
Apolipoprotein B	apoB	FPEVDVLTK
		TEVIPPLIENR
Apolipoprotein C-I	apoC1	TDVSSALD
		EWFSETFQK
Apolipoprotein C-II	apoC2	TAAQNLYEK
		TYLPAVDEK
Apolipoprotein C-III	apoC3	DALSSVQESQVAQQAR
		GWVTDGFSSLK
Apolipoprotein E	apoE	LGPLVEQGR
		AATVGSLAGQPLQER
Apolipoprotein J (Clusterin)	CLUS	TLLSNLEEAK
		IDSLLENDR
Hemoglobin alpha chain	HBA	VGAHAGEYGAELER
		FLASVSTVLTSK
Human serum albumin	HSA	DDNPNLPR
		LVNEVTEFAK

For a subset of MACE intermediate biomarkers, in-depth proteomics (>3500 peptides and >450 proteins on a Q-Exactive-HFX (Thermo Fisher Scientific, San Jose, CA) and lipidomics (1153 lipids on the Lipidyzer™ platform, SCIEX) will be performed on plasma samples obtained at enrollment and exit. A targeted lipid panel representing 13 lipid classes (Table 3) will be analyzed using the Lipidyzer™ platform (SCIEX) which consists of a triple quadrupole mass spectrometer (5500 Q-trap) with a SelexION™ front end.^3^ The SelexION™ technology maximizes the correct identification of isobaric lipids, thus adding an extra level of specificity. The system has a validated kit composed of multiple internal standards specifically for each lipid class to allow for accurate quantification and eliminate quantitative bias. Plasma lipids will be extracted, processed with internal standards based on the manufacturer’s protocol, and analyzed by direct infusion from the auto sampler.^17^ Lipid concentrations will be determined by the Lipidyzer software using the ratio of the endogenous lipid to internal standard.

**Table 3:** Lipid classes analyzed by the Lipidyzer^TM^

Lipid class	Number of lipid species
Triacyclglycerols (TAG)	502
Diacylglycerols (DAG)	67
Free Fatty Acids (FFA)	28
Cholesterol Esters (CE)	34
Phosphatidylcholines (PC)	161
Phosphatidylethanolamines (PE)	233
Lysophosphatidylcholines (LPC)	28
Lysophosphatidylethanolamines (LPE)	28
Sphingomyelins (SM)	16
Ceramides (CER)	56
Total	1153

### Patient reported outcomes (PROs)

In addition to monitoring biochemical biomarkers and wearable biometrics, we will complement these data streams with patient-reported outcomes (PROs) collected through a mobile phone application or web browser using HealthLoop, an electronic platform capable of administering surveys to patients and facilitating remote patient monitoring in the form of dashboards and communication tools for health professionals. Patients will receive an email reminder the week prior to complete the forms through push notification if the HealthLoop application installed on their smartphones. PROs to be collected include the following short-form subscales of the PROMIS^®^ questionnaire:^18^ depression (eight items), emotional distress/anxiety (eight items), fatigue (eight items), physical function (12 items), sleep disturbance (eight items), social isolation (eight items), global mental health (four items), and global physical health (four items). The PROMIS questionnaire subscales query participants about the previous 7 days and will be delivered each week for the duration of the study. Participants will receive the 12-item Kansas City Cardiomyopathy Questionnaire (KCCQ-12) every other week to generate a cardiomyopathy summary score and assess cardiomyopathy-specific physical limitation, symptom frequency, quality of life, and social limitations.^19^ Participants will receive the seven-item Seattle Angina Questionnaire (SAQ-7) every 30 days to create an angina summary score and angina-specific physical limitations, symptom frequency, and quality of life.^20^ At the exit, participants will be asked about each of the platforms used in a perceived ease of use survey to evaluated patient engagement and experience.^21^

### Follow up and major adverse cardiovascular events

MACE is defined as all-cause mortality, myocardial infarction, stroke, and heart failure hospitalizations. Secondary MACE includes any cardiovascular hospitalization, including revascularization, other treatment and observation for suspected acute coronary syndrome, transient ischemia attack, or arrhythmia. Participants will be followed at the 1-year time point by phone to assess for MACE outcomes that may have occurred after the monitoring period. If the 1-year follow-up survey is incomplete, the Social Security Death Registry will be accessed for mortality information.

### Data integration

Data from baseline and exit surveys will be collected in Research Electronic Data Capture (REDCap) to preserve protected health information.^22^ Data from Fitbit Charge 2 devices will be collected in real-time and synced sporadically with the application installed on participant's smartphones via Bluetooth. A research-grade service (Fitabase, San Diego, CA) will be used to retrieve minute-level data from Fitbit servers from all participants. In addition to retrieving raw step counts and heart rates each minute, the service provides epoch-level wear-time and classification of activity into sedentary, light, moderate, and vigorous activity levels using proprietary and validated algorithms. The data will be maintained at minute-level epochs and merged to lower resolutions (e.g., day-level, week-level) for specific data analyses. Data from the AliveCor KardiaMobile device will be received as post-processed time-stamped recordings with diagnostic information that is merged as a count of use and count of confirmed atrial fibrillation weekly. Access to readings of raw ECG and voice recordings will occur on a case-by-case basis, for the purposes of case studies and qualitative analysis. Data from PROs will be uploaded to HealthLoop after each survey is completed and scores will be downloaded at the end of the participants’ enrollment. The Health Measures Scoring Service will be used to score PROMIS surveys in order to use population-based *t*-scores, per scoring manual specifications.

Lower limits of quantification and detection for all biomarker data will be obtained and reported. Data analysis for the LC-MS/MS apolipoprotein panel data will be performed with MultiQuant^™^ 3.0 software (SCIEX) for the 6500 triple quadrupole mass spectrometers and using Pinnacle (Optys Technologies, Boston, MA). The peak area ratio of each endogenous peptide relative to their stable-isotope labeled analog will be calculated. Protein results are reported as the average, normalized, peak area ratio from one selected proteotypic peptide, whereas the second proteotypic peptide is used for confirmation. The lipid concentrations obtained from the Lipidyzer software will be used to determine differential lipid profiling of lipids species and classes.

## Plan of analysis

### Aim 1 Goal: to measure cross-sectional correlations between remote monitoring data and established MACE intermediate biomarkers, including hsCRP, NT-proBNP, and u-hs-cTnI

To accomplish cross-sectional analyses, we will first calculate Pearson correlations to describe the bivariate relationships between each daily-averaged wearable metric (mean daily steps, miles, calories, active movement, sleep hours, sleep interruptions, heart rate) over the first week and the first week PRO metrics (PROMIS^®^ physical function, fatigue, anxiety, depression, sleep disturbance plus SAQ and KCCQ), each versus baseline MACE intermediate biomarkers and apolipoproteins.

The study will then use generalized linear models (GLMs) to describe the baseline relationships between both monthly- and weekly-averaged metrics (PROMIS, SAQ, KCCQ, and ambulatory physiological measures) with baseline MACE intermediate biomarkers (hsCRP, NT-proBNP, u-hs-cTnI) and apolipoproteins. Models will be evaluated for assumptions and data will be transformed as needed, including standardization to aid in interpreting results. GLMs will adjust for relevant covariates including, but not limited to, age, sex, ethnicity, income, and other comorbidities. Process models such as hidden Markov models will be used to model the temporal dynamics of biosensor data over the course of the study. These models will be combined with machine learning classifiers such as random forests and support vector machines to predict patient outcomes such as changes in MACE intermediate biomarkers and PROs. We hypothesize that subtle but detectable clinical parameters from remote patient monitoring data and PRO data will correlate with MACE intermediate biomarkers.

### Aim 2 Goal: to measure the longitudinal ability of remote monitoring data to predict changes in MACE intermediate biomarkers

We will use generalized linear mixed models (GLMMs) to describe the baseline and exit relationships between both monthly- and weekly-averaged metrics and baseline MACE intermediate biomarkers and apolipoproteins. Violations of assumptions in models will be handled with transformation, as needed. The GLMMs will be used to evaluate the longitudinal analyses examining the relationship between time-varying ambulatory physiological measurements (e.g., steps), weekly and monthly PROs, and time-varying intermediate biomarkers. GLMMs will be adjusted for covariates. We hypothesize that changes in remote patient monitoring data and PRO data will predict changes in MACE intermediate biomarkers.

### Exploratory Aim 2b: to measure the ability of remote monitoring with PROs, wearable biometrics, and traditional biomarkers to predict incident MACE at baseline and over 12 months of patient follow-up

We anticipate that 10–13% of participants will develop MACE during the 12-month follow-up. This event rate is limited to fully power the study for MACE as the primary outcome. As an exploratory aim, we will use wearable biometrics, PROs, MACE intermediate biomarkers and apolipoproteins, plus discovery protein and lipid panels and run data models to identify multivariable predictors of MACE on a subset of MACE intermediate biomarkers. Through this discovery proteomic and lipidomic panels, over 450 additional proteins and 1153 lipids representing a broad systemic response, to inflammation, vascular reactivity, extra cellular matrix disturbance, kidney function will be explored. Limits to develop an exploratory composite MACE prediction index using multivariate modeling will also be implemented. We will exploit the large-scale multi-omic data to define potentially underlying molecular mechanisms of MACE through three methods: (1) using existing knowledge bases to provide functional annotation, molecular pathways, and protein interacting partners; (2) using text mining platforms to extract information from the literature to elucidate links/relationships to other health conditions and cardiac phenotypes; (3) creating a test dataset first and then using supervised and unsupervised learning models to understand multi-omic data relevancy and ability to predict features of cohort subsets. Using this data, we will run Cox proportional hazard models to identify multivariable predictors of MACE. Due to the rarity of MACE, this model may yield infinite estimates; in that event, we will prepare an alternative model using the Firth–Cox method. The discriminative power of the final multivariable MACE prediction composite score will be assessed by area under the receiver operating characteristic curve (C-Statistic). By combining PROs, remote patient monitoring data, and at-home monitoring of known MACE biomarker predictors from Aims #1–2 plus >500 additional blood proteins representing a broad systemic response, such as inflammation (e.g., serum amyloid A), vascular reactivity (e.g., periostin), extra cellular matrix disturbances (e.g., MMPs), kidney function (e.g., cystatin C), and lipids (e.g., omega fatty acids), we will develop an exploratory composite MACE prediction index using multivariable modeling.

### Aim 3 Goal: to estimate the cost-effectiveness and budget impact of remote monitoring for MACE

Digital health innovations should be subjected not only to analysis of efficacy and effectiveness, but also to analyses of cost-effectiveness. In this protocol, we will use results from the study to create hypothesis-generating cost-effectiveness and budget impact models to estimate the projected costs per life year saved, and per-member per-month cost of remote monitoring vs. usual care. Models will feature a time horizon of 5 years and employ Markov-chains with 1-month cycles. We will use state transition probabilities derived from a systematic literature review, and supplement these with remote monitoring prediction estimates derived from data collected in the study. Estimates of direct health care costs will assume the health-system perspective and include immediate and downstream costs associated with disease progression. Costs of the remote monitoring will be assessed via a micro-costing and will also account for ongoing support of particpants by incorporating staff salaries in the health economic calculation. Finally, we will conduct deterministic and probabilistic sensitivity analyses using one-way, two-way, and Monte Carlo approaches. We hypothesize that incremental cost of remote monitoring will be offset by downstream savings engendered by early and precise prediction of unexpected and costly MACE in stable moderate-risk IHD.

### Reporting summary

Further information on research design is available in the Nature Research Reporting Summary linked to this article.

## Supplementary information

Reporting Summary

## Data Availability

The datasets generated during and/or analyzed during the current study are available from the corresponding author on reasonable request.

## References

[1] Benjamin EJ (2018). Heart disease and stroke statistics-2018 update: a report from the American Heart Association. Circulation.

[2] Stone AA, Broderick JE, Junghaenel DU, Schneider S, Schwartz JE (2016). PROMIS fatigue, pain intensity, pain interference, pain behavior, physical function, depression, anxiety, and anger scales demonstrate ecological validity. J. Clin. Epidemiol..

[3] Ashok V, Melody T, Michele A, Steve A (2017). Remote patient monitoring via non-invasive digital technologies: a systematic review. Telemed. e-Health.

[4] Pevnick JM, Fuller G, Duncan R, Spiegel BMR (2016). A large-scale initiative inviting patients to share personal fitness tracker data with their providers: initial results. PLOS ONE.

[5] Braunwald E (2008). Biomarkers in heart failure. N. Engl. J. Med..

[6] van den Broek I (2017). Application of volumetric absorptive microsampling for robust, high-throughput mass spectrometric quantification of circulating protein biomarkers. Clin. Mass Spectrom..

[7] Dorresteijn JA (2013). High-dose statin therapy in patients with stable coronary artery disease: treating the right patients based on individualized prediction of treatment effect. Circulation.

[8] Benedetto S (2018). Assessment of the Fitbit Charge 2 for monitoring heart rate. PLOS ONE.

[9] Zide M (2018). Evaluating utility and compliance in a patient-based eHealth study using continuous-time heart rate and activity trackers. J. Am. Med. Inform. Assoc..

[10] Kociol RD (2010). Troponin elevation in heart failure: prevalence, mechanisms, and clinical implications. J. Am. Coll. Cardiol..

[11] Evans GF, Shirk A, Muturi P, Soliman EZ (2017). Feasibility of using mobile ECG recording technology to detect atrial fibrillation in low-resource settings. Glob. Heart.

[12] Jarolim P (2015). Fully automated ultrasensitive digital immunoassay for cardiac troponin I based on single molecule array technology. Clin. Chem..

[13] Rissin DM (2010). Single-molecule enzyme-linked immunosorbent assay detects serum proteins at subfemtomolar concentrations. Nat. Biotechnol..

[14] Blood collection devices. https://www.neoteryx.com/micro-sampling-capillary-blood-collection-devices. Accessed 15 Oct 2018.

[15] Fu Q (2018). Highly reproducible automated proteomics sample preparation workflow for quantitative mass spectrometry. J. Proteome Res..

[16] van den Broek I, Sobhani K, Van Eyk JE (2017). Advances in quantifying apolipoproteins using LC-MS/MS technology: implications for the clinic. Exp. Rev. Proteom..

[17] Bligh EG, Dyer WJ (1959). A rapid method of total lipid extraction and purification. Can. J. Biochem. Physiol..

[18] Bevans M, Ross A, Cella D (2014). Patient-Reported Outcomes Measurement Information System (PROMIS): efficient, standardized tools to measure self-reported health and quality of life. Nurs. Outlook.

[19] Spertus JA, Jones PG (2015). Development and validation of a short version of the Kansas City Cardiomyopathy Questionnaire. Circ. Cardiovasc. Qual. Outcomes.

[20] Chan PS, Jones PG, Arnold SA, Spertus JA (2014). Development and validation of a short version of the Seattle Angina Questionnaire. Circ. Cardiovasc. Qual. Outcomes.

[21] Davis FD (1989). Perceived usefulness, perceived ease of use, and user acceptance of information technology. MIS Q..

[22] Harris PA (2009). Research electronic data capture (REDCap)—a metadata-driven methodology and workflow process for providing translational research informatics support. J. Biomed. Inform..

